# High magnetic sensitivity via large-diameter-vortex stability in magnetic tunnel junctions through controlled anisotropy

**DOI:** 10.1103/41by-5p3c

**Published:** 2025-09-23

**Authors:** Benjamin J. Brown, Liam K. Mitchell, Vineetha S. Bheemarasetty, H. Minh Cao, Justin N. Kingsnorth, Jerome N. Sanes, Gang Xiao

**Affiliations:** 1 Department of Physics, Brown University, Providence, Rhode Island 02912, USA; 2 Department of Neuroscience and Carney Institute for Brain Science, Brown University, Providence, Rhode Island 02912, USA; 3 Center for Neurorestoration and Neurotechnology, Veterans Affairs Providence Healthcare System, Providence, RI 02908, USA

## Abstract

Magnetic-annealing-induced uniaxial anisotropy is shown to influence vortex formation in magnetic tunnel junction (MTJ) free layers of various diameters, with micromagnetic simulations demonstrating a threshold for single-vortex formation that exhibits an inverse diameter dependence. Angular *M*-*H* curves were measured for MTJ multilayers under different annealing conditions, and reveal that a two-step orthogonal magnetic annealing process reduces the uniaxial anisotropy in the free layer from 1040 J*/*m^3^ to 130 J*/*m^3^. This reduction in anisotropy allows for vortex formation in MTJ sensors as large as 40 μm. Increasing the MTJ diameter leads to an enhancement of magnetic sensitivity up to 2.80%*/*Oe with the conventional definition, or 1.78%*/*Oe via an ac sensitivity measurement that verifies the nonhysteretic response. This result demonstrates an eightfold increase in vortex-MTJ sensitivity, compared with standard MTJs that have diameters limited to 5 μm, and this work provides the foundation for highly sensitive vortex-MTJ-based sensors for applications that require a nonhysteretic, ultrasensitive response.

## I. INTRODUCTION

Magnetic tunnel junctions (MTJs) have emerged as a leading spintronic technology for magnetic field sensing applications due to their high sensitivity [1–3], low power consumption [4–6], low noise [7,8], and extended temperature stability [9–12]. These versatile sensors are used in various applications, including biomedical imaging [13,14], navigation, and nondestructive testing [15,16]. Conventional MTJ sensors, while delivering excellent performance at higher magnetic fields, suffer from reduced sensitivity, undesirable nonlinearity, and measurement errors at lower fields due to hysteresis. This challenge has prompted extensive research into various mitigation techniques [17–20]. In contrast, vortex-MTJ sensors offer an inherently nonhysteretic response, making them attractive for low-field applications [21,22]. These sensors face geometrical constraints on their thickness [23] and diameter, which limit the ability to achieve high sensitivity. This limitation stems from the requirement to maintain a stable vortex configuration within the free layer [24].

Researchers have explored various methods to improve the sensitivity of vortex MTJs. One technique involves implementing a composite free layer with a magnetic material that has lower inherent anisotropy [25]. This anisotropy reduction pushes the maximum diameter for stable vortex formation from 5 μm to 9 μm. This approach yields a minor sensitivity increase, but has the advantage of simpler fabrication. Another proposed method is to engineer the geometry of the pinned layer such that the diameter is smaller than the free layer [26]. This results in the vortex core saturating prematurely and causes the sensor to be highly responsive to external field. This technique shows a sizable increase in sensitivity, but requires precise patterning using e-beam lithography. In this work, we propose a technique that achieves high sensitivity while preserving the straightforward and simple fabrication process of a typical MTJ.

Building upon recent works utilizing two-step annealing to improve linearity and reduce hysteresis [27,28], we employ a similar technique and demonstrate its effectiveness in reducing uniaxial anisotropy in the free layer. Reducing the anisotropy is key for linearizing the sensor response, as well as promoting the formation of a stable vortex state even in larger-diameter MTJ devices. Larger-diameter vortex MTJs exhibit reduced dynamic range and increased sensitivity, which is why we aim to increase the diameter of the stable vortex phase. We show through simulation that a reduction in free layer anisotropy enables the reemergence of the vortex state in devices with diameters up to 40 μm, and experimentally demonstrate this by fabricating devices with reduced anisotropy using a two-step orthogonal annealing process.

## II. EXPERIMENTAL

The MTJ multilayer, deposited onto thermally oxidized silicon wafer substrates by dc and rf magnetron sputtering, consisted of Ta(5)/Ru(20)/Ta(5)/Ru(2)/Ir_22_Mn_78_ (18)/Co_50_Fe_50_(3)/Ru(0.85)/Co_40_Fe_40_B_20_(3.5)/MgO(2.8)/Co_40_Fe_40_B_20_(55)/Ta(7)/Ru(5) (thicknesses in nanometers) [29]. The magnetic layers below the MgO (CoFe-RuCoFeB) layer are antiferromagnetically coupled and serve as a magnetically compensated reference layer [30]. Above MgO, the 55-nm CoFeB free layer is in the thickness range to support vortex formation. Further details regarding this stack can be found in our previous works [1,15,31].

The multilayer was micropatterned into circles with diameters ranging from 10 to 40 μm. Physical ion milling was used to etch below the MgO layer. Following this, the devices were passivated with SiO_2_, and gold leads were deposited for contacts using e-beam evaporation, resulting in two same-diameter MTJs in series. Additionally, 4-mm-diameter circles and 50 × 50 arrays of 40-μm circles were fabricated with the same multilayer for vibrating sample magnetometry (VSM) measurements. A high-vacuum magnetic annealing was performed at 360 °C in a field of 0.45 T, with a ramp rate of 6 °C*/*min followed by a 60-min soak at the target temperature. Several temperatures and durations were tested to optimize this procedure, leading to tunnel magnetoresistance (TMR) ratios of approximately 125%. Subsequent lower-temperature, orthogonal annealings performed under the same conditions resulted in slightly decreased TMR ratios attributed to further boron and interfacial diffusion.

Magnetization data of the 4-mm-diameter circles and 40 μm diameter arrays were collected using a MicroSense Inc. VSM. A Micromanipulator VERSA probe station equipped with two-axis Helmholtz coils, capable of both ac and dc applied fields, was used for the magnetotransport measurements. These measurements were taken with an applied voltage of approximately 100 mV to the MTJs under test. The direct sensitivity of the two-MTJ device was measured by applying an ac modulated 1-Oe field (rms) at 40 Hz and measuring the voltage response with a lock-in amplifier. The small ac magnetic field was super-imposed on a slowly varying dc magnetic field to measure the sensitivity as a function of the dc bias field.

Micromagnetic simulations were conducted using MUMAX3, a GPU-accelerated program [32,33], varying diameter and uniaxial anisotropy parameters. The sweep range was selected to ensure full sensor saturation. Thermal noise was not included in the simulations. All other parameters were held constant: cell size = 3 nm, thickness = 60 nm, saturation magnetization *M*_*s*_ = 800 emu/cm^3^, exchange stiffness *A*_ex_ = 13 pJ/m [34,35], and damping constant *α* = 0.004 [36].

## III. RESULTS AND DISCUSSIONS

High-temperature magnetic annealing was performed in a strong magnetic field to define the magnetic sensing direction in our MTJs and to crystallize the MgO and CoFeB interface, allowing for high TMR ratios via coherent tunneling [37]. This process induces uniaxial anisotropy along the magnetic field direction through a mechanism distinct from magnetocrystalline anisotropy. At elevated temperatures, increased atomic diffusivity in CoFeB enables atoms to migrate in response to the field. Variations in different atom interaction energies drive a subtle, field-aligned lattice distortion that becomes kinetically trapped upon cooling. This directional anisotropy, or easy axis, contributes to the free layer’s magnetic energy and is central to enhancing vortex stability in this study.

The magnetic vortex state, a distinct magnetic domain configuration, forms under specific conditions to minimize the total magnetic energy, which is made up of several contributing interactions. This energy can be expressed as

(1) $$E=E_{\text{ex}}+E_{\text{ani}}+E_{\text{demag}}+E_{Z}.$$

Here, *E*_ex_ denotes the exchange energy, favoring parallel alignment of neighboring spins. The anisotropy energy, *E*_ani_, represents the energy cost for the magnetization direction to deviate from the easy axis. *E*_demag_, the magnetostatic or demagnetization energy, accounts for the energy associated with magnetic poles that generate external fields. Finally, the Zeeman energy, *E*_Z_, describes the interaction of magnetic moments with an external magnetic field. In the simulations, we sweep the external field, but determine the ground state domain structure when *E*_Z_ = 0.

When exchange energy and anisotropy dominate, a single-domain state forms within the free layer. However, this state generates magnetic dipoles along the easy axis, contributing to *E*_demag_. If the energy associated with these poles exceeds the combined exchange and anisotropy energies, the magnetostatic energy becomes dominant. In this case, the magnetic energy is minimized when the spins align tangentially around a core, effectively eliminating uncompensated poles. This process is referred to as vortex-state nucleation, achievable only in smooth, continuous geometries like circles and ellipses.

Between the single-vortex and single-domain states, an intermediate state containing two vortex cores exists. These cores partially increase the magnetostatic energy associated with poles relative to a single vortex. However, due to each of the cores exhibiting opposite chiralities, a significant portion of the spins align parallel, reducing exchange energy. The boundaries defining the energetic favorability of each of these states remain unclear.

A systematic study of the preferred magnetic domain state as a function of *E*_ani_ and *E*_demag_ was performed using micromagnetic simulations to characterize this phase space. *E*_ani_ was varied by changing the anisotropy constant *K*_*u*_, while *E*_demag_ was varied by changing the free layer diameter, which changes the pole separation and hence the strength of the demagnetizing field. To determine the stable zero-field ground state without influence from a predefined state, each simulation was initialized with a randomized spin configuration, and allowed to relax to a minimum-energy state. Subsequently, a full hysteresis loop was simulated by applying an external field sufficient for saturation. The magnetic state at zero field in each sweep direction was then determined through direct visualization of the domains and categorized.

**FIG. 1.**
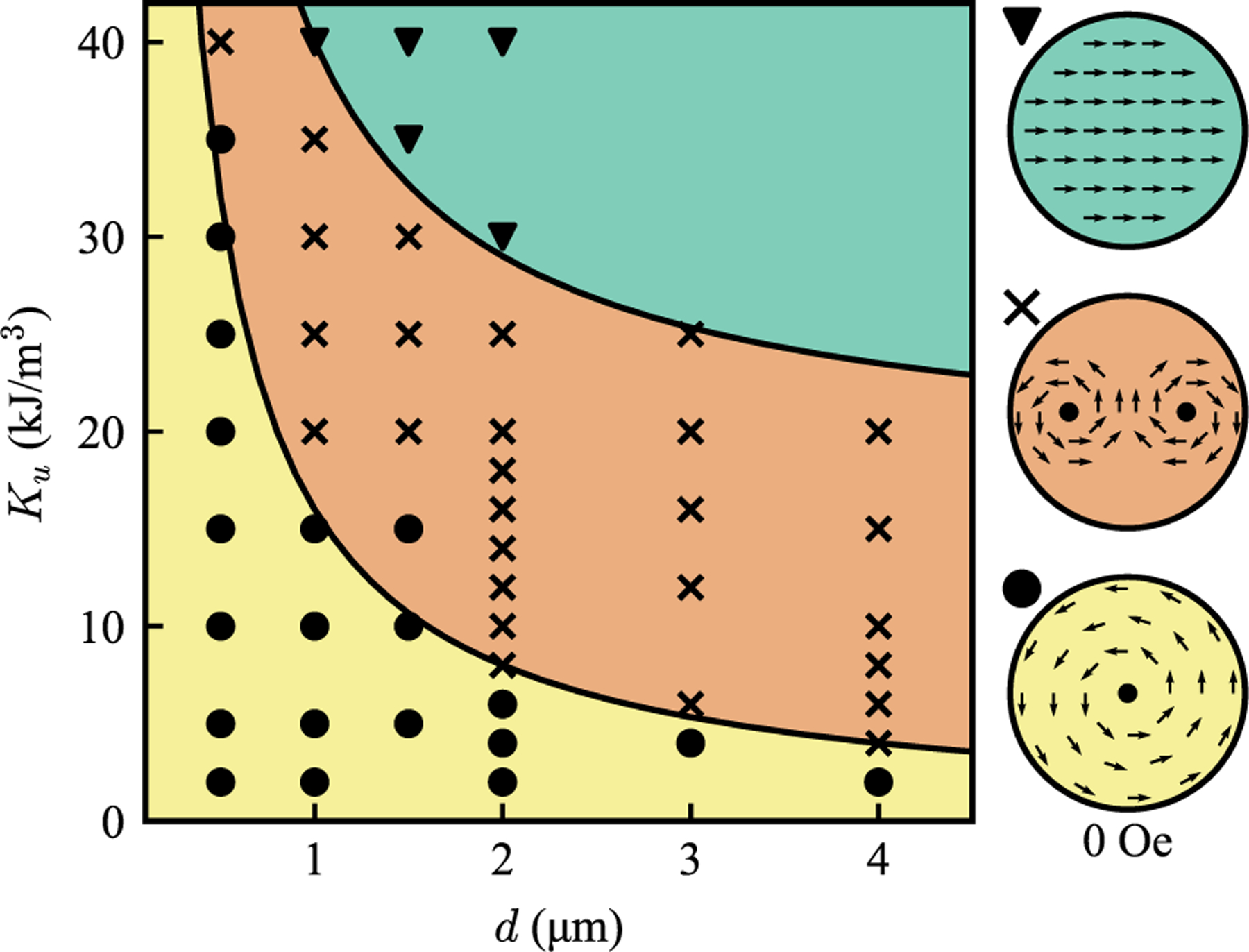
Phase diagram depicting the zero-field magnetic domain state of the free layer as a function of uniaxial anisotropy and MTJ diameter, *d*. The single-vortex (yellow), double-vortex (orange), and single-domain (green) states are observed with increasing diameter or anisotropy, with the single vortex threshold obeying a 1*/d* trend.

Figure 1 illustrates this phase space representation of the zero-field magnetic domain states. Due to the computational demands of simulating MTJs with larger diameter (a 40-μm free layer would take months on a top-performing graphics card), this study focused on 0.5–4-μm-diameter MTJs with anisotropy constants, *K*_*u*_, ranging from 0 to 40 kJ*/*m^3^. The upper range exceeds values typically induced via magnetic annealing (about 1 kJ*/*m^3^), but is required to explore the full phase space in smaller MTJs. Although simulations were limited to smaller diameters, they capture the key interplay between diameter, anisotropy, and domain structure. The underlying principles of micromagnetic energy minimization support extrapolation to larger devices, as the same competing energies govern domain formation across larger scales. This is further validated by our experiments, where anisotropy reduction, consistent with the simulated trend, stabilizes vortex states in large MTJs.

We observed the three distinct magnetic domain states in the simulated free layers: first, a single-vortex state with a nonhysteretic core exhibiting a linear response to external perturbations, which is the desired configuration for sensor applications; second, a double-vortex state, characterized by two cores of opposite chirality, exhibiting a hysteretic response to an applied magnetic field; and finally, a single-domain uniaxial state, exhibiting a characteristic square *M*-*H* loop. Representative *M*-*H* curves for each state are shown in Appendix B. The hysteresis found in the latter two states make them unsuitable for sensing applications, therefore Fig. 1 defines the threshold conditions for stable single-vortex formation as a function of anisotropy and diameter.

The results presented in Fig. 1 are consistent with the energy minimization principles outlined in Eq. (1). In small-diameter MTJs, the presence of strong, closely spaced magnetic poles leads to a dominant magnetostatic energy, favoring the removal of the poles over the exchange energy, and forms a vortex state. With a constant anisotropy, increasing the diameter reduces the magnetostatic energy, allowing the exchange energy to dominate and promote a transition back to the uniaxial state. Similarly, keeping diameter constant and increasing the anisotropy energy also favors the uniaxial state by effectively reinforcing the exchange energy.

These simulations revealed an empirical relationship clearly defining regions for each magnetic domain phase. The boundary between the single-vortex and double-vortex states follows a 1*/d* relationship, where *d* is the diameter of the MTJ. This scaling implies that for vortex devices with larger diameters, the permissible anisotropy for maintaining the single-vortex state is lower than the anisotropy induced via annealing. Consequently, the free layer may transition into a hysteretic state.

This insight reveals the central strategy of our work: to intentionally reduce the uniaxial anisotropy, enabling large-diameter MTJ vortex devices that exhibit high sensitivity. The magnetic annealing process, being the primary source of this anisotropy, is therefore the essential tool that must be engineered to achieve this reduction.

**FIG. 2.**
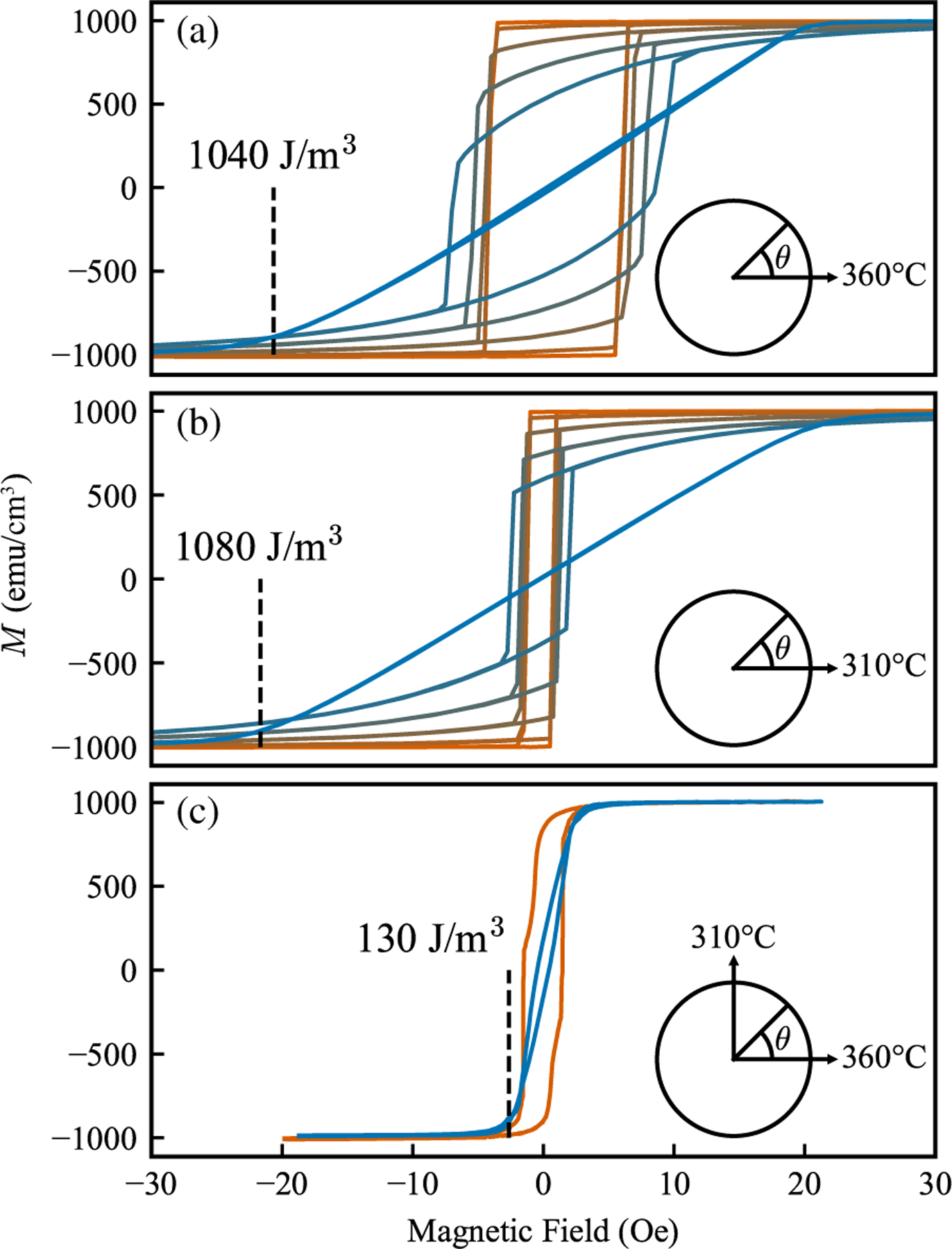
In-plane experimental *M*-*H* curves showing angular major loops (hysteresis loops measured at different in-plane angles) for 4-mm circular samples. Angles relative to the initial annealing field direction from 0° (orange) to 90° (blue) are shown, with intermediate angles and colors between. Every 15° is shown, omitting 75° for clarity due to overlap with other curves. The inset diagrams explain the magnetic annealing temperature and directions, which are: (a) 360 °C single annealing; (b) 310 °C single annealing; (c) 360 °C annealing, followed by a 310 °C annealing orthogonal to the first. (Only 0° and 90° shown.)

*M*-*H* measurements were performed on 4-mm-diameter disks, varying the sample angle relative to the initial annealing direction. Due to their small thickness-to-diameter ratio, these samples exhibit negligible demagnetization effects [38]. Samples that were annealed once demonstrate a clear and well-defined easy axis, evident in the square hysteresis loop shown in Figs. 2(a) and 2(b). Magnetic reversal in these bulk samples occurs through domain wall displacement. Along the easy axis, the applied field opposes the annealing-induced magnetization. Upon reaching the coercive field, cascading domain wall motion leads to complete saturation in the opposite direction. Conversely, applying the field along the hard axis results in a gradual rotation of the magnetization away from the easy axis. The saturation field in this direction is directly pro-portional to the anisotropy constant. The other curves show intermediate angles that exhibit mixed easy- and hard-axis features.

To determine the uniaxial anisotropy constant, we first extracted the saturation field, *H*_*k*_. This was done by finding the intersection of a line fitting the linear portion of the hard-axis (90°) curve to another line fitting the saturated regime. The uniaxial constant, *K*_*u*_, can then be calculated using *H*_*k*_ and the saturation magnetization, *M*_*s*_, using the equation

(2) $$K_{u}=\frac{H_{k}M_{s}}{2}.$$

The calculated *K*_*u*_ values at each corresponding *H*_*k*_ are noted in Fig. 2.

The initial magnetic annealing process induces a uniaxial anisotropy of 1040 J*/*m^3^ in the MTJ free layer. Lowering the annealing temperature to 310 °C does not significantly alter the induced anisotropy, although the coercive field along the easy axis is substantially reduced. This can be attributed to a decrease in the magnetic domain grain size resulting from the lower temperature conditions [39].

Subsequent annealing processes with the magnetic field *orthogonal* to the initial direction lead to an eightfold reduction in the anisotropy. This observation can be explained by considering the mechanism of annealing-induced anisotropy. Elevating the sample temperature to a level sufficient for atomic mobility, but lower than the initial annealing temperature, allows atoms to partially reorient with the new field direction. Consequently, the sequential orthogonal annealings effectively compensate each other, removing the slight lattice distortion that caused the anisotropy, while preserving the desired crystalline barrier.

The ability to reduce the uniaxial anisotropy by an order of magnitude using orthogonal annealing directly enables access to the low-*K*_*u*_ regime predicted in Fig. 1 for stable vortex formation in large devices. We now investigate how this anisotropy tuning translates to real device performance.

**FIG. 3.**
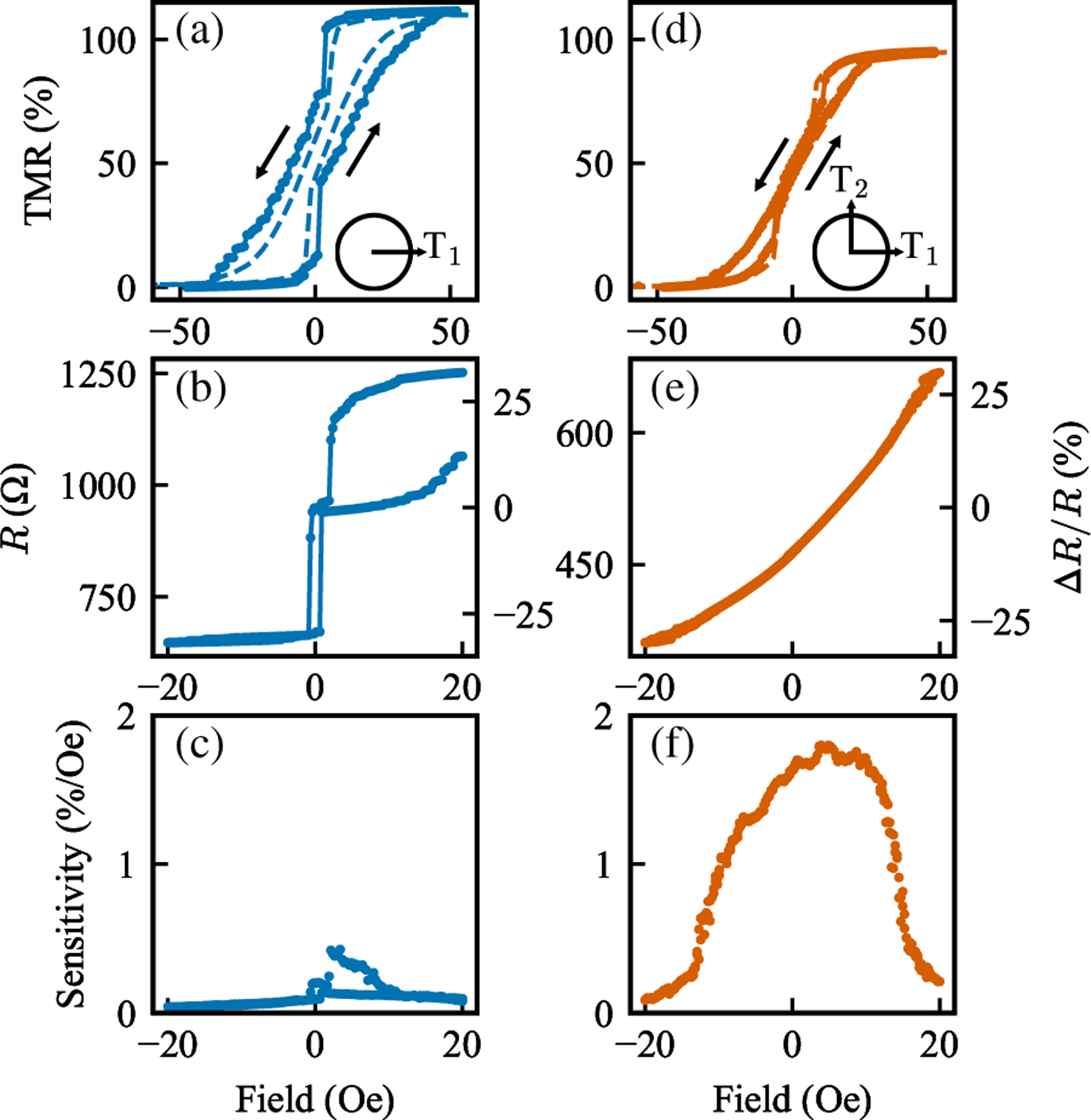
Effect of orthogonal annealing on magnetoresistance and sensitivity measurements in 40-μm MTJs. After the first annealing: (a) Full loop showing hysteresis, indicating anisotropy is higher than the single-vortex threshold. The dotted line shows the magnetic VSM response normalized to the TMR. (b) Minor loop confirming irreversibility of the single annealed sensor. (c) Direct ac sensitivity measurement, obtained by applying a small ac magnetic field and measuring the resulting change in voltage. After the second, orthogonal annealing and reduction in anisotropy: (d) Nonhysteretic vortex state, demonstrating the formation of a stable, single-vortex state. (e) Minor loop, showing no hysteresis, confirming the stability of the single-vortex state. (f) Direct ac sensitivity measurement, showing a significant improvement in sensitivity compared to the single-annealed case.

To test this experimentally, we fabricated 40-μm MTJs and measured their transport properties before and after the two-stage annealing treatment. Figure 3 illustrates how the reduction in anisotropy modifies the magnetoresistive response, enabling a transition from a hysteretic state to a robust single-vortex configuration in large-diameter devices.

The initial 360 °C annealing induces a uniaxial anisotropy of 1040 J*/*m^3^ in the free layer, as confirmed by the VSM measurements. According to micromagnetic simulations, this level of anisotropy exceeds the thresh-old for stable single-vortex formation in large-diameter MTJs. This is reflected experimentally in Figs. 3(a)–3(c), where the full magnetoresistance loop exhibits hysteresis, the minor loop is erratic and nonreversible, and the ac sensitivity is both weak and hysteretic.

Following a second, orthogonal annealing at 310 °C, the anisotropy is reduced to 130 J*/*m^3^, which is within the simulated regime for stable vortex states. The resulting transport characteristics, shown in Figs. 3(d)–3(f), demonstrate a clear transition to the desired behavior. The full loop is hysteresis-free with a vortex shape, the minor loop becomes linear and reversible, and the ac sensitivity significantly improves, reaching up to 1.78%/Oe without hysteresis. Minor loops and ac sensitivity measurements for devices of other diameters are shown in Appendix A.

The ac sensitivity shown in Fig. 3(f) confirms this, showing a significantly enhanced sensitivity of 1.78%*/*Oe. The ac sensitivity, *S*_ac_, was measured by applying a small ac field, Δ*H*, and measuring the resulting change in response, Δ*V*, relative to the device bias voltage, *V*. *S*_ac_ is defined as

(3) $$S_{\text{ac}}=\frac{\Delta V}{V\Delta H}.$$

This measurement more accurately captures the performance of the device, as it directly measures the output signal in response to a magnetic field perturbation, which closely resembles the sensor’s operation in practical applications.

**FIG. 4.**
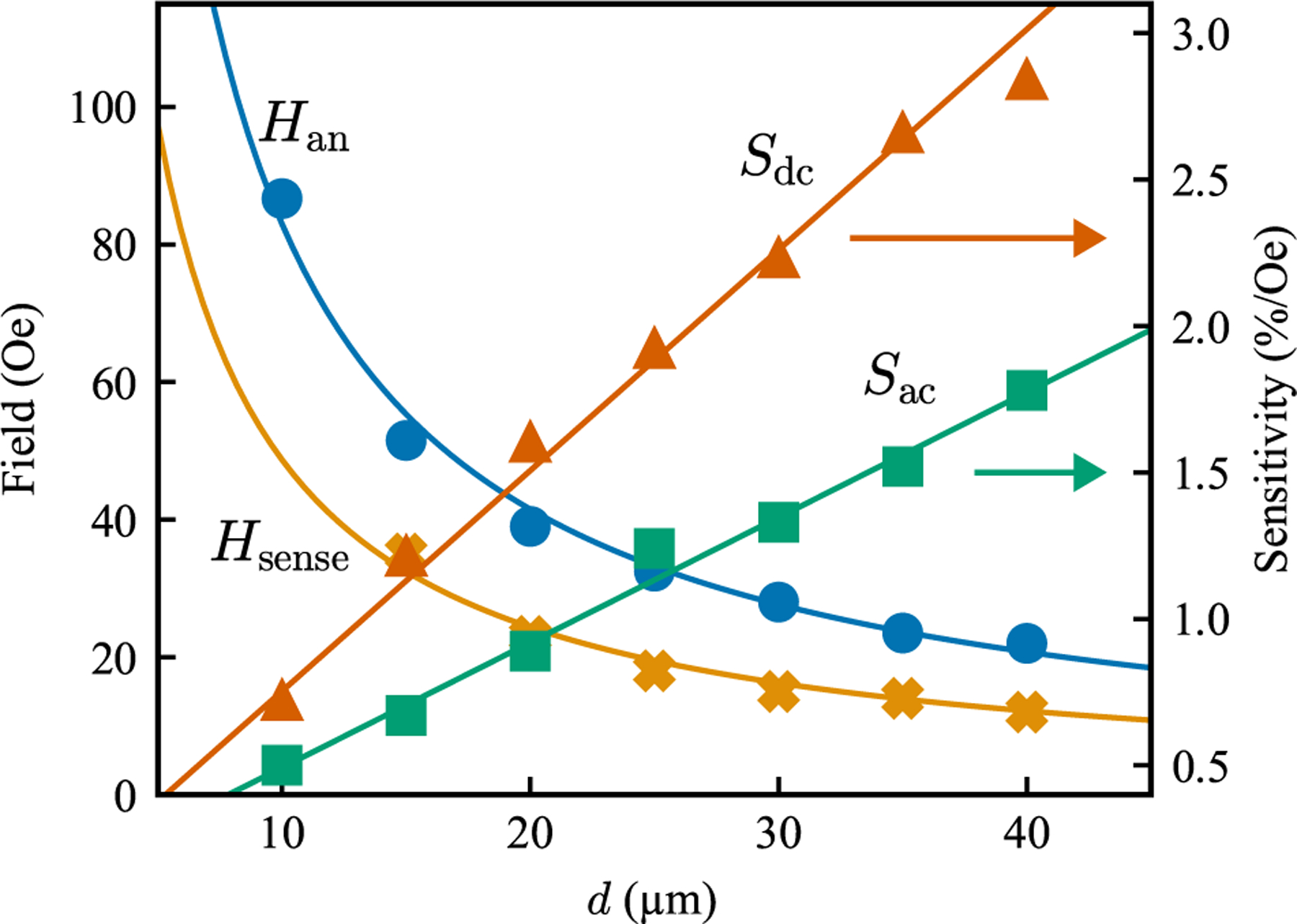
A summary of the experimentally measured sensitivity and dynamic range results for MTJs of varying diameters. *H*_an_ denotes the annihilation field and *H*_sense_ is the ac sensitivity dropoff field, characteristic of the magnetic dynamic range. *S*_ac_ and *S*_dc_ are the ac and dc sensitivities, as defined in Eqs. (3) and (4).

Finally, we extend this approach across a range of device diameters to quantify the impact of vortex stability on sensor sensitivity. As Fig. 4 shows, the two-stage annealing enables vortex formation at larger diameters, yielding a systematic enhancement in both ac and dc sensitivity.

Sensitivity was increased and dynamic range was reduced, and the annihilation field, *H*_an_, for each diameter is also shown. *H*_an_ is used to estimate the sensitivity using

(4) $$S_{\text{dc}}=\frac{\text{TMR}}{2H_{\text{an}}}.$$

The calculated dc sensitivity using an approximate TMR of 125% is presented in the figure. To better represent the sensor’s operational range, we define *H*_sense_ as the field at which the ac sensitivity drops to half of its maximum value. This *H*_sense_ was approximately 58% of *H*_an_, providing a relevant metric for application-specific evaluations. Finally, the ac sensitivity, which linearly increases with device diameter, is shown.

Table I highlights several techniques that aim to improve sensitivity by reducing dynamic range. Each result has been normalized by its respective TMR value, allowing for a comparison of sensor effectiveness independent of device barrier quality. Techniques involving magnetic flux concentrators are omitted, as they amplify the external field and are not characteristic of the MTJ sensor itself.

Compared to other common approaches, our method achieves comparable or enhanced normalized sensitivity while maintaining a simple and scalable fabrication process. Unlike high-aspect-ratio MTJs, which often exhibit small but nonzero hysteresis, our vortex-based design is intrinsically hysteresis-free. This is especially important for sensing low external fields, where even minimal hysteresis can degrade sensitivity. Furthermore, the use of larger diameters not only facilitates vortex stability but is also expected to improve noise performance, as magnetic noise scales inversely with the volume of the free layer. These combined features, including zero hysteresis, improved noise characteristics, and ease of fabrication, make our approach well suited for robust sensing devices and high-density sensor arrays.

## IV. CONCLUSION

In this study, we explored the effects of a two-step orthogonal annealing process on the magnetic properties and sensor performance of MTJ devices. Our findings demonstrate that this technique effectively suppresses uniaxial anisotropy in the free layer, enabling the formation of stable vortex states in larger-diameter MTJs. This transition to a vortex configuration in the large-diameter regime is crucial for achieving nonhysteretic sensing, as it mitigates the constraints imposed by traditional shape anisotropy. By reducing hysteresis and enhancing low-field response, this approach broadens the design space for highly sensitive vortex-based MTJ sensors.

By facilitating vortex formation in MTJs with diameters up to 40 μm, we achieved a substantial improvement in sensor performance, with sensitivity reaching 1.78%/Oe in ac measurements and 2.80%/Oe using the conventional dc definition—while maintaining a nonhysteretic response. This represents an eightfold increase compared to conventional MTJs with smaller 5 μm diameters. In ultralow-field sensing applications, achieving a low dynamic range with zero hysteresis is crucial for minimizing signal distortion and ensuring high-fidelity detection. Furthermore, the increased sensor volume associated with larger diameters helps suppress magnetic noise, improving signal stability and overall sensitivity. Finally, our two-step annealing process enables tunable sensitivity and dynamic range, providing flexibility in optimizing sensor performance for specific applications.

## Figures and Tables

**TABLE I.** Comparison of *S*_ac_ and *S*_dc_ [as defined in Eqs. (3) and (4)] of various techniques, normalized by the TMR of each respective study.

MTJ Technique	*S*_dc_ (%/Oe)	*S*_ac_ (%/Oe)	Limitation
Conventional vortex-based [40]	0.26	0.30	Limited sensitivity
Vortex composite free layer [25]	0.45	0.35	Limited sensitivity
Large aspect ratio [27]	0.85	…	Residual 3.5% hysteresis
Nonorthogonal annealing [41]	1.26	…	Restricted dynamic range
Vortex double annealing (this work)	2.24	1.40	Compromised spatial resolution
Vortex pinned layer geometry [26]	3.16	…	Requires complex fabrication (e-beam lithography)

**FIG. 5.**
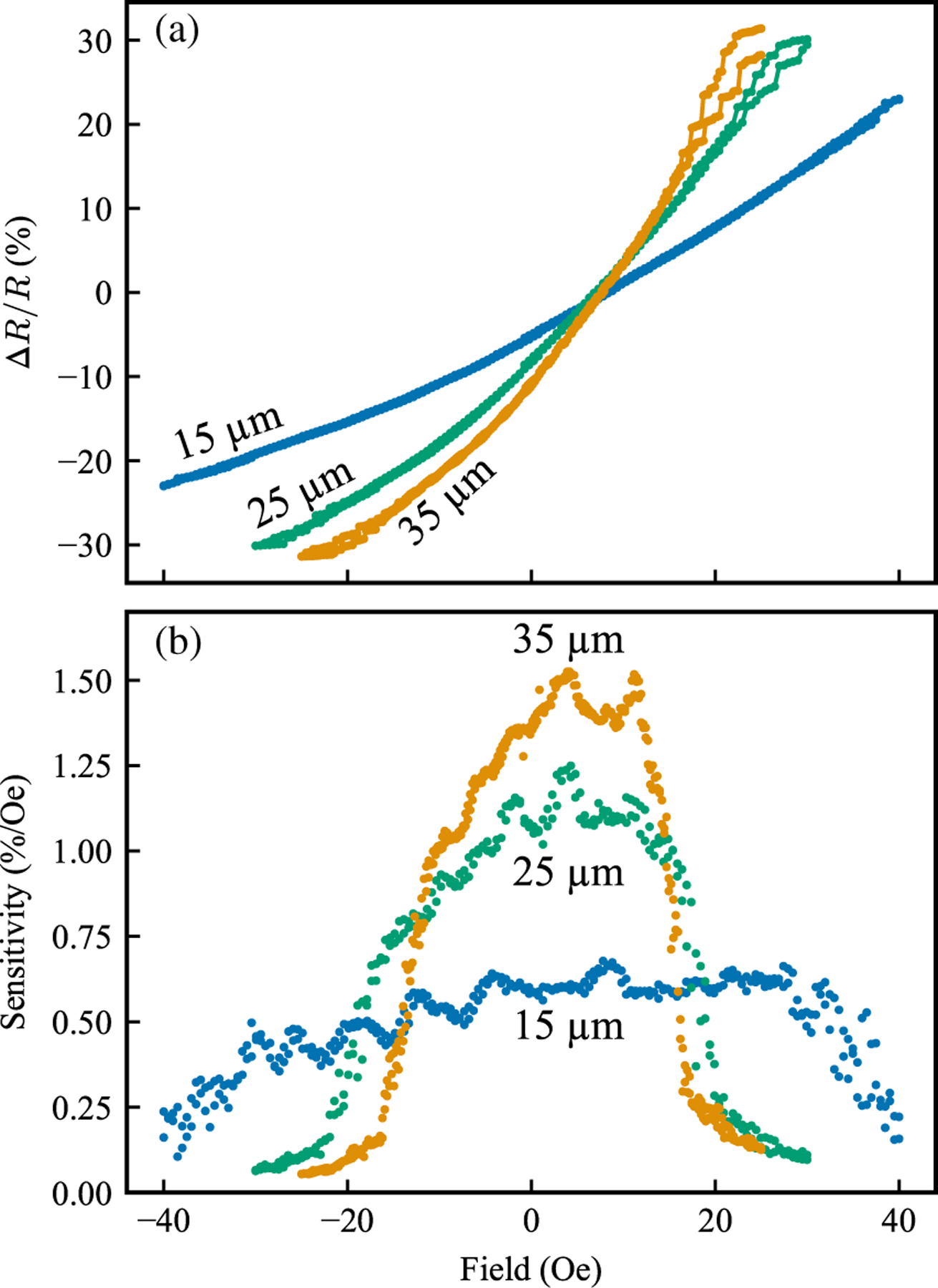
Normalized resistance minor loops and ac sensitivity measurements for devices with intermediate diameters. (a) Minor loops showing no hysteresis, confirming the stability of the single-vortex state. (b) Direct ac sensitivity measurements, showing the true sensitivity and response of the sensors.

**FIG. 6.**
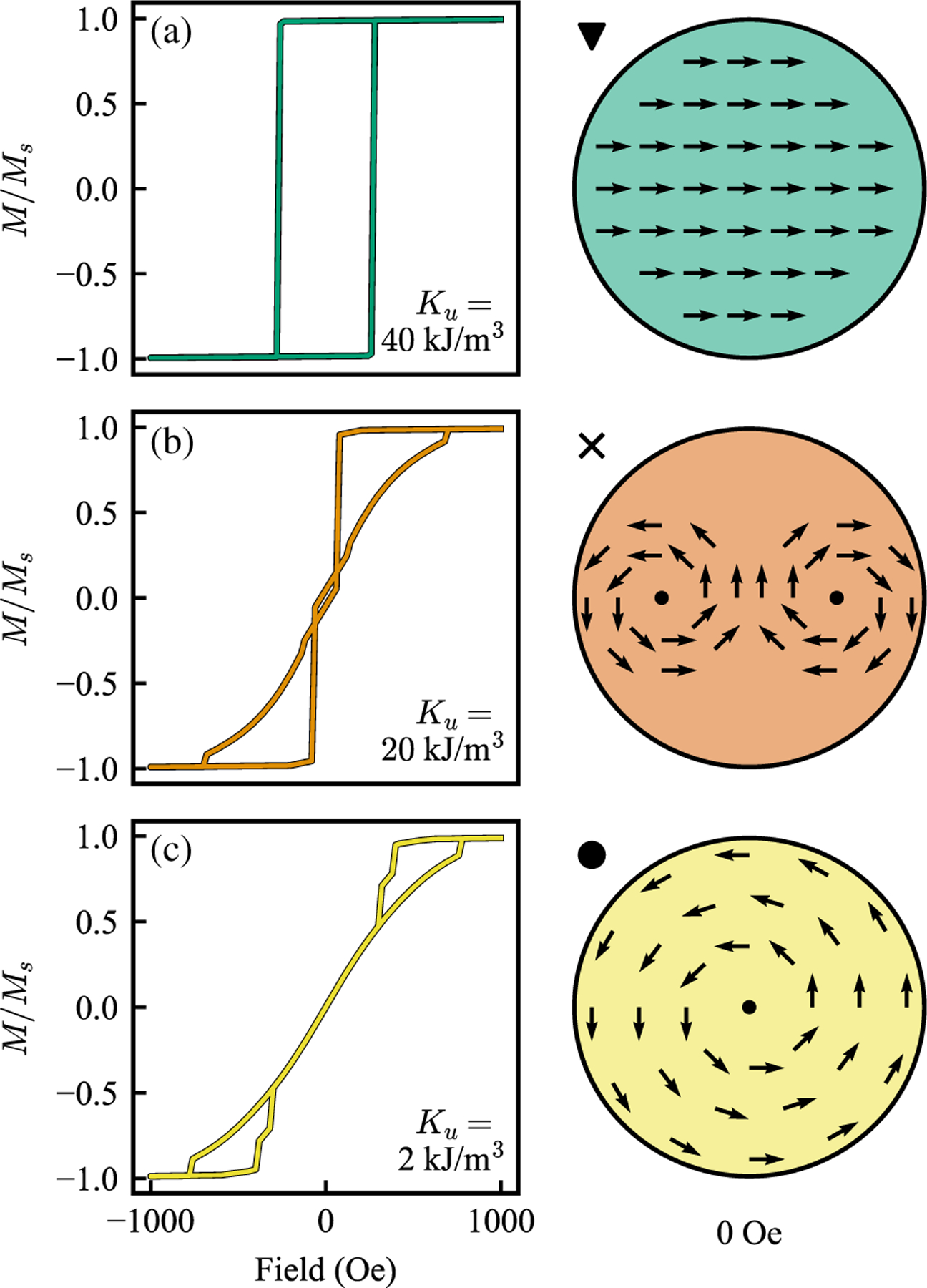
Simulated magnetization loops for a 1-μm-diameter magnetic free layer. (a) Single-domain state. (b) Double-vortex state. (c) Single-vortex devices, the desired configuration.

## Data Availability

The data that support the findings of this article are openly available [42].

## APPENDIX A: INTERMEDIATE-DIAMETER RESULTS

The orthogonal double-annealing technique demonstrated in this work was also applied to devices of various sizes below the 40-μm example shown in Fig. 3. Consistent behavior was observed across devices ranging from 10 to 40 μm, where the single-vortex, nonhysteretic state is recovered. Representative results for selected sizes are shown in Fig. 5. These devices were annealed under the same conditions as the one discussed earlier: an initial annealing at 360 °C, followed by a second, orthogonal annealing at 310 °C.

## APPENDIX B: SIMULATED *M*-*H* LOOPS

Figure 1 presents simulation results across a range of device diameters and anisotropy constants. Each simulation was categorized based on the magnetic domain configuration at zero field during the field sweep. Representative simulated *M*-*H* curves and domain visualization at zero field for each domain classification are shown in Fig. 6. These loops correspond directly to the points in Fig. 1 for a 1-μm device at each respective anisotropy value.
